# Putting the pH into phosphatidic acid signaling

**DOI:** 10.1186/1741-7007-9-85

**Published:** 2011-12-02

**Authors:** John JH Shin, Christopher JR Loewen

**Affiliations:** 1 Department of Cellular and Physiological Sciences, Life Sciences Institute, University of British Columbia, 2350 Health Sciences Mall, Vancouver, BC, Canada, V6T 1Z3

## Abstract

The lipid phosphatidic acid (PA) has important roles in cell signaling and metabolic regulation in all organisms. New evidence indicates that PA also has an unprecedented role as a pH biosensor, coupling changes in pH to intracellular signaling pathways. pH sensing is a property of the phosphomonoester headgroup of PA. A number of other potent signaling lipids also contain headgroups with phosphomonoesters, implying that pH sensing by lipids may be widespread in biology.

## Review

**Figure 1:**
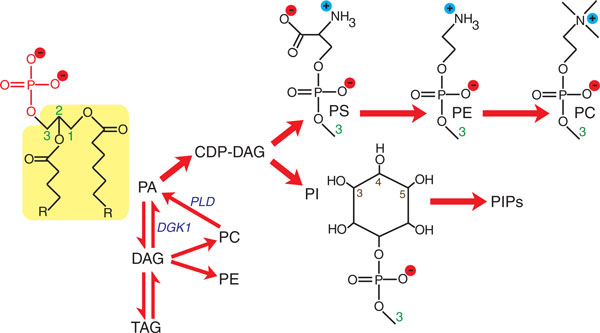
**Phosphatidic acid is a key precursor in lipid metabolism**. A simplified outline of the major lipids originating from phosphatidic acid (PA) is shown. These include glycerophospholipids: CDP-diacylglycerol (CDP-DAG), phosphatidylserine (PS), phosphatidylethanolamine (PE), phosphatidylcholine (PC), phosphatidylinositol (PI), and phosphoinositol phosphates (PIPs); and neutral lipids: diacylglycerol (DAG) and triacylglycerol (TAG). PA is drawn in its deprotonated form carrying a charge of 2-. Its structure is composed of a phosphomonoester headgroup (shown in red) attached to a DAG backbone (highlighted in yellow). This DAG backbone is composed of glycerol with two acyl chains attached at its *sn1 *and *sn2 *positions (green numerals indicate the *sn *positions of glycerol, R represents the remaining structure of each acyl chain that is not shown). All glycerophospholipids have their headgroups attached to the DAG backbone at the *sn3 *position of glycerol. The headgroups of PS, PE, PC and PI are shown for comparison and the positions of phosphorylation of the inositol ring in PIPs are labeled (3,4,5). The actions of DAG kinase (DGK) and phospholipase D (PLD) in regulating PA levels are also indicated (shown in blue italics).

Lipids are best known for their structural role in forming lipid bilayers in cells, which facilitates inter- and intracellular compartmentalization. However, prominent roles for several lipids in signal transduction have also emerged in the past decade [1-3]. Phosphatidic acid (PA) is an anionic lipid consisting of a negatively charged phosphomonoester headgroup attached to a hydrophobic diacylglycerol backbone (Figure 1). It is present in all organisms and serves as a key intermediate in the synthesis of neutral lipids (di- and triacylglycerol) and all glycerophospholipids [4-6]. These include phosphatidylserine (PS), phosphatidylethanolamine (PE), phosphatidylcholine (PC), phosphatidylinositol (PI), and the phosphatidylinositol phosphates (PIPs), which together make up the bulk of cellular membranes (Figure 1) [4,5]. In addition, PA is emerging as an important signaling lipid.

**Figure 2:**
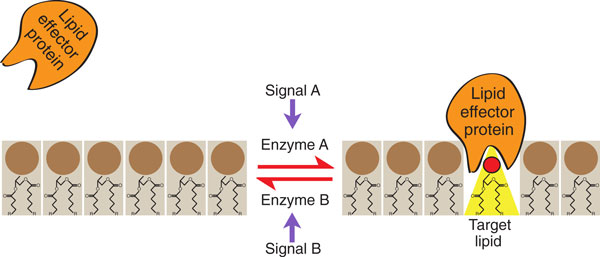
**Lipid signaling is concentration dependent**. Lipid effector proteins are unable to bind membranes when the concentration of their target lipid is low (left). Cellular signals lead to the activation of lipid-modifying enzymes, which generate target lipids that can then recruit the effector proteins to the membrane (right). Conversely, other cellular signals can activate enzymes that convert the target lipid back to its original form or to another lipid, causing release of the effector from the membrane. In this example, the target lipid highlighted in yellow is also cone-shaped (for example, PA, PE or DAG) in contrast to the majority of membrane lipids, which are cylindrical in shape (for example, PC and PS; headgroups colored brown). The conical shape reduces packing of lipid headgroups, which exposes the hydrophobic acyl layer surrounding the target lipid. This may facilitate insertion of hydrophobic amino acids and effector binding.

Signaling lipids in general are thought to act by binding effector proteins and recruiting them to a membrane, which regulates the proteins' activity in cellular pathways. Binding is primarily dependent on the concentration of the lipid in the bilayer. Changes in lipid concentration induced by lipid-modifying enzymes in response to upstream signals generate downstream signaling responses (Figure 2) [3]. For PA, its concentration is maintained at low levels in the cell as a result of its continuous conversion into other lipid species, which balances its *de novo *synthesis [4,5]. For this reason PA makes up only around 1% of total cellular lipid content [7]. However, PA can also be produced from lipid reserves, such as through the hydrolysis of PC by phospholipase D (PLD) and the phosphorylation of diacylglycerol (DAG) by DAG kinase (DGK), and these routes have important signaling roles (Figure 1) [3].

PLD activation is the best-characterized route by which cells generate PA in signaling responses [8,9]. In mammalian cells, PLD is a potent regulator of the Ras signaling pathway, which is strongly implicated in cancer and well known to regulate cell proliferation, differentiation and apoptosis [10,11]. In this case, stimulation by epidermal growth factor results in increased activity of PLD at the plasma membrane and generation of localized PA at this site [10]. Generation of PA leads to the binding and recruitment of the effector protein Son of sevenless (Sos) to the plasma membrane, which leads to Ras activation [10]. Similarly, the kinase Raf-1 is activated by PA generated by PLD, which results in activation of downstream mitogen-activated protein kinase signaling [12-14]. In addition, PA can bind and activate the mammalian target of rapamycin (mTOR), a protein kinase well known for its roles in cell survival and cancer [15,16]. This activation by PA has also been linked to PLD regulation [17]. Sphingosine kinase, which has important roles in cell growth, calcium homeostasis and cell movement [18], is also regulated by PLD- and PA-dependent recruitment to intracellular membranes [19]. In yeast, PA signaling is required for sporulation, the yeast equivalent of gametogenesis [20,21]. PLD is responsible for generating PA during this event, which activates the membrane recruitment of the SNARE protein Spo20, which in turn drives formation of the prospore membrane [20-22].

PA signaling has also been strongly implicated in numerous other cellular processes, including vesicular trafficking, cytoskeletal dynamics and stress responses [3,23]. Despite the detailed characterization of the many pathways regulated by PA, the mechanisms underlying its binding to effector proteins are not well understood. An important question is how do proteins recognize PA over other abundant anionic lipids, such as PS and PIPs. A second and related question is how is PA in a particular membrane recognized over PA in another, as the lipid is present in most intracellular membranes at similar concentrations, but binding of effectors appears to be membrane specific [12,24]. Answering these questions is critical to understanding the regulation of PA signaling and is of clinical importance because of emerging roles for PA and its protein effectors in disease, especially cancer [16,17,25]. In this review, we will discuss these questions by introducing how charge and pH govern effector binding and how PA signaling is regulated by intracellular pH.

## Interaction of proteins with phosphatidic acid

We will start by discussing how PA interacts with its effector proteins and how specificity is likely to be achieved. PA effectors are different from other lipid-binding proteins because they lack an obvious conserved primary amino acid sequence [23,26]. Instead, binding to PA is generally specified through nonspecific electrostatic interactions between clusters of positively charged amino acids in the protein and the negatively charged phosphomonoester headgroup of PA [14,20,24,26-28]. In addition, most PA-binding domains also contain interspersed hydrophobic residues that are thought to facilitate membrane association through their insertion into the bilayer [26,29]. PS is another negatively charged lipid that is nearly 10-fold more abundant than PA in cells and is the predominant anionic species in the plasma membrane, comprising around 34% of total plasma membrane phospholipids; and PS effectors also employ similar binding strategies [7,26]. Yet rarely do PA effectors also bind PS. A key question, therefore, is how is specificity for PA achieved?

**Figure 3:**
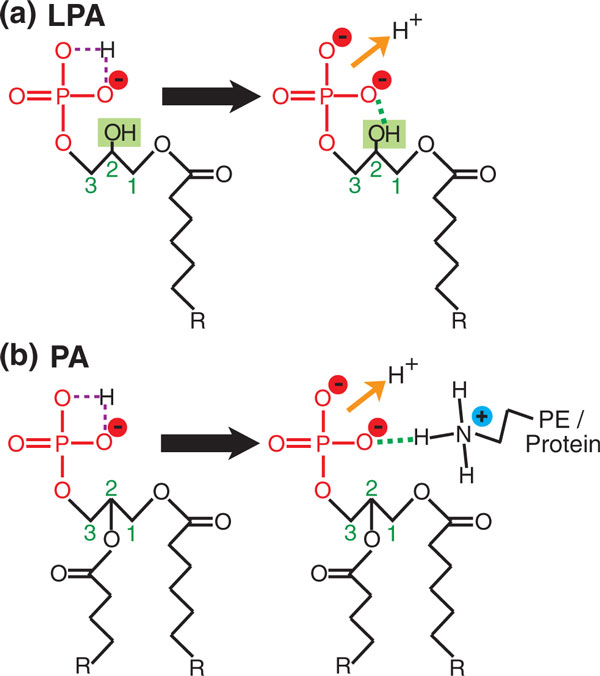
**Hydrogen bonding increases the charge of phosphatidic acid (PA) and lysophosphatidic acid (LPA)**. **(a) **The phosphomonoester headgroup of LPA forms an intramolecular hydrogen bond. In the protonated phosphomonoester of LPA (left) a proton is shared between two hydroxyl oxygens (purple dashed lines). Hydrogen bonding (green dashed lines) between the *sn2 *hydroxyl and the phosphomonoester of LPA competes with the shared proton for oxygen electrons, which facilitates dissociation of the proton lowering the p*K_a_*. **(b) **Hydrogen bonding between the phosphomonoester of PA and the primary amine of the headgroup of PE, or lysines and arginines of proteins, results in deprotonation of the phosphomonoester, lowering its p*K_a _*and increasing its negative charge. The abundance of PE in cellular membranes is likely a significant factor regulating the p*K_a _*of both PA and LPA [30]. Hydrogen bonding between proteins and PA is described by the electrostatic/hydrogen bond switch mechanism [29].

PA is unique among phospholipids in that it contains a phosphomonoester headgroup rather than the phosphodiesters in other phospholipids such as PS, in which the phosphate group is also linked to a serine (Figure 1). Work by Kooijman *et al. *[27] revealed that the charge state of the phosphomonoester in PA, compared to that of other anionic lipids, is key to achieving specificity in effector binding. Kooijman *et al. *[30] first shed light on the nature of PA recognition by using ^31^P-NMR to study the ionization state of PA and lysophosphatidic acid (LPA) in model membrane bilayers. LPA, like PA, has a phosphomonoester headgroup, but differs in that its second acyl chain is replaced by a hydroxyl group (Figure 3a). Kooijman *et al. *discovered that LPA has a significantly lower p*K_a _*than PA (7.5 for LPA compared with 7.9 for PA) and therefore carries significantly greater negative charge at physiological pH [30] (p*K_a _*is the negative logarithm of the ionization constant of an acid). This difference was attributed to hydrogen bonding between the phosphomonoester of LPA and the hydroxyl group replacing its missing acyl chain (Figure 3a). Interestingly, inclusion of PE, another excellent hydrogen-bond donor, in model membranes lowered the p*K*_*a *_values of both PA and LPA even further, to the same value of 6.9. On this evidence, the authors proposed a model in which hydrogen bonding of the effector protein to the phosphomonoester of PA enables it to carry a greater negative charge at physiological range [30]. Hydrogen bonding between the hydroxyl oxygens of PA and hydrogen-bond donors, such as the primary amines of PE, results in destabilization of the remaining proton of the phosphate, probably as a result of increased competition for oxygen electrons (Figure 3b). This facilitates the dissociation of this proton, which ultimately increases the negative charge of PA.

Lysines and arginines in proteins also represent an excellent source of hydrogen-bond donors because of their primary amines. Therefore, a key question was whether hydrogen bonding between the phosphomonoester of PA and lysines and arginines in PA-binding domains had a role in binding specificity. Kooijman *et al. *[29] demonstrated that small peptides composed of lysine and arginine residues also increase the charge of PA, probably as a result of hydrogen bonding. This culminated in the proposal of the 'electrostatic/hydrogen-bond switch mechanism', which provides an elegant explanation of how PA effectors specifically recognize PA [29]. In this model a PA-binding protein is initially attracted to a negatively charged membrane bilayer as a result of electrostatic interactions. The protein then randomly samples the bilayer until it encounters protonated PA, which possesses a charge of 1-. Once in contact, the basic amino acids of this protein hydrogen bond with the phosphomonoester of PA to cause dissociation of its remaining proton (Figure 3b). This results in a switch in charge from 1- to 2-, which strengthens the electrostatic interaction and locks the protein onto deprotonated PA. This unique property explains the specificity of effectors for PA over other negatively charged lipids such as PS. The latter has a phosphodiester with a maximum charge of 1- (Figure 1) that does not change over the physiological range, making electrostatic/hydrogen-bond switching irrelevant. Thus, PA effectors favor binding to PA over PS because of the higher negative charge of PA and the stronger electrostatic interactions.

The role of arginine as a hydrogen-bond donor in PA-effector interaction is exemplified by work on the FKBP12-rapamycin binding (FRB) domain of mTOR, one of the few characterized PA-binding domains to have been co-crystallized with PA. Veverka *et al. *[31] crystallized the FRB domain bound to soluble PA containing short acyl chains. From this crystal structure, it was found that a patch of only five solvent-exposed amino acids were active in PA docking. Of these, only a single basic amino acid, an arginine, was absolutely required for binding. This agrees with the original characterization of PA binding to mTOR, where mutation of this arginine significantly inhibited PA binding [15]. Veverka *et al. *also showed that the arginine is in close proximity to the headgroup of PA and forms a positively charged patch, which the phosphomonoester interacts with. This close proximity should therefore allow hydrogen bonding between the primary amine of arginine and the phosphomonoester of PA, in agreement with the electrostatic/hydrogen-bond switch model [29]. Furthermore, the PA-binding site in the FRB domain is a shallow pocket that is also lined with hydrophobic residues that would be able to penetrate into hydrophobic insertion sites surrounding PA [31]. The shallowness of the pocket also implies that lipids with bulkier headgroups - for example, PS and PIPs - would encounter steric hindrance, which would not be the case for PA.

Lastly, the small cross-sectional area of the phosphomonoester headgroup of PA relative to the DAG backbone gives PA a cone-shaped structure (Figure 2), making PA the only cone-shaped anionic lipid in the cell and distinguishing it yet further from PS, which is a cylindrically shaped lipid [32,33]. The cone shape prevents tight packing of PA's headgroup with the headgroups of neighboring lipids, which exposes the hydrophobic acyl layer of the bilayer surrounding PA. This loose packing provides an excellent site for insertion of hydrophobic amino acids of PA effectors, which facilitates their binding to the membrane (Figure 2) [32,33]. For example, PA stimulates penetration of dynamin into the acyl layer of membranes even though dynamin does not directly bind PA [34].

PE is also a cone-shaped lipid, but differs from PA in that it is neutral at physiological pH [32]. As part of their work on PA effector specificity, Kooijman *et al. *[29] found that incorporation of PE into PA-containing liposomes enhanced specific binding of Raf kinase to PA. A role for PE in PA-effector binding was also demonstrated by Young *et al. *[28] for binding of a transcriptional repressor in yeast, Opi1, which will be discussed in more detail in the next section. As we saw earlier, one reason for the enhancement in binding by PE could be through increasing the charge on PA by its role as a hydrogen-bond donor (Figure 3b); but its conical shape may also facilitate the bilayer-insertion of hydrophobic residues of PA effectors, which also contribute to binding. PS, in contrast, because of its cylindrical shape, does not facilitate binding in this way. Together, all these findings indicate that effector recognition of PA is achieved through a combination of electrostatic/hydrogen-bond switching and the availability of hydrophobic insertion sites in the bilayer surrounding PA.

## Phosphatidic acid is a pH biosensor in yeast

There are two main reasons to suspect that PA could act as an intracellular pH sensor. First, the p*K_a _*of the phosphomonoester headgroup is within physiological range (6.9 to 7.9) [30]. The p*K_a _*is the negative logarithm of the ionization constant (K) of an acid and is equal to the pH at which half of the acid molecules are ionized. For example, if the p*K*_*a *_of PA is equal to 7, at pH 7 it will be 50% deprotonated. This means that 50% of the PA molecules will carry a net charge of 1- and 50% will be 2-. If the pH changes one pH unit in either direction, to pH 6 or pH 8, PA will be approximately 90% protonated or approximately 90% deprotonated, respectively. Thus, because of its p*K_a_*, PA is poised to change its protonation state maximally in response to physiological changes in intracellular pH. Second, the electrostatic/hydrogen-bond switch mechanism predicts that PA effectors will have higher affinity for deprotonated over protonated PA. Thus, protein effectors have the capacity to detect changes in the levels of deprotonated PA in response to changes in pH, which cells can then exploit in signaling pathways. Together, these features suggest that PA is a pH biosensor.

Such a role for PA was recently shown by Young *et al. *[28] through a genome-wide screen in yeast to identify new regulators of PA signaling. In yeast, PA in the endoplasmic reticulum regulates expression of genes controlling phospholipid synthesis and lipid metabolism. It does this through binding and sequestering a transcriptional repressor, Opi1, outside the nucleus on the cytoplasmic leaflet of the endoplasmic reticulum. When PA in the endoplasmic reticulum is depleted, Opi1 is released and translocates to the nucleus, where it coordinately represses transcription of more than 30 genes, thus enabling global repression of lipid metabolism [24,35]. *INO1 *is the most highly regulated of these genes and is responsible for the synthesis of inositol, which is critical for the cell [35]. Hence, any defect in binding between Opi1 and PA results in the constant repression of *INO1 *and the inability to synthesize inositol. This makes inositol auxotrophy (the inability to grow in medium lacking inositol) an ideal screening phenotype for finding mutants with dysregulated PA signaling [28,36].

**Figure 4:**
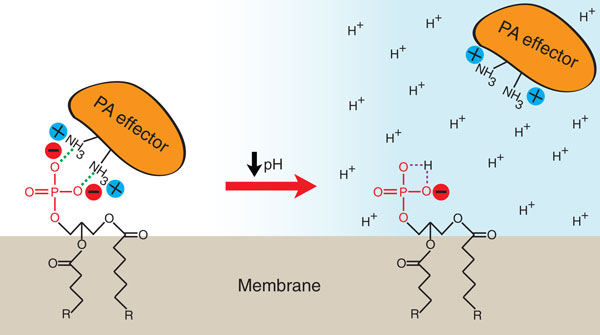
**Phosphatidic acid is a pH biosensor**. Effector proteins bind PA according to the electrostatic/hydrogen-bond switch mechanism [29]. Electrostatic interactions and hydrogen bonding (green dashed lines) between the primary amines of basic amino acids in the effector and the deprotonated phosphomonoester of PA are shown. A decrease in intracellular pH below the p*K_a _*of PA results in increased protonation of its phosphomonoester, which reduces the strength of electrostatic interactions with the effector, resulting in its dissociation from the membrane.

Young *et al. *[28] discovered that mutants with defects in the regulation of cytosolic pH were highly enriched in their inositol auxotrophy screen. The genes involved included those for both the major regulators of pH in yeast - the plasma membrane proton ATPase (Pma1) and the vacuolar proton ATPase (V-ATPase) [37]. Pma1 functions as the master regulator of cytosolic pH in yeast by pumping protons produced by glycolysis in the cytosol out of the cell. In this role, Pma1 consumes nearly 20% of total cellular ATP and is the most abundant plasma-membrane protein in yeast [38,39]. The V-ATPase also contributes to cytosolic pH regulation by pumping protons from the cytosol into the vacuole and by regulating trafficking of Pma1 to the plasma membrane [37]. Thus, mutants defective in either Pma1 or V-ATPase activity are sensitive to acidification of the cytosol [37]. The normal cytosolic pH of yeast cells is rigorously maintained at a pH around 7.2, despite sometimes harshly acidic extracellular conditions [39,40]. Young *et al. *exploited a hypomorphic mutant of Pma1 that was incapable of maintaining physiological pH under conditions of extracellular acid stress and showed that cytosolic acidification correlated with repression of lipid metabolism genes. This was due to the release of Opi1 from the endoplasmic reticulum under conditions of cytosolic acidification. They also showed that binding of Opi1 to PA *in vitro *was dependent on pH, where binding decreased with acidification from a pH of around 7 to one of about 6. Using a non-titratable methylated derivative of PA, Young *et al. *[28] demonstrated that the pH effect on binding was due to a change in the protonation state of PA. Thus, Opi1 has greater affinity for deprotonated PA at higher pH, which is consistent with the electrostatic/hydrogen-bond switch model (Figure 4).

A remaining question was the physiological function of pH-sensing by PA. Yeast are highly sensitive to glucose availability in their environment because it is their preferred energy source [41]. One response to glucose withdrawal is cytoplasmic acidification, which results from the rapid inactivation of both Pma1 and the V-ATPase [37,38,42]. Young *et al. *[28] found that Opi1 was released from the endoplasmic reticulum and translocated to the nucleus upon glucose starvation. Opi1 translocation was dependent on cytoplasmic pH and correlated with a drop below a pH of around 6.9, consistent with the decreased affinity of Opi1 for protonated PA. Thus, PA is a pH biosensor that coordinates nutrient sensing and cell-growth signaling by regulating the synthesis of new membranes.

## pH sensing by phosphatidic acid in animal cells

The appropriate cytosolic pH must be rigorously maintained in all cells, not just those of yeast, because of the massive generation of protons and other metabolic acids by core metabolic processes [39,43]. Although pH-sensing by PA in yeast has now been demonstrated, a key question is how relevant are lipid pH biosensors to signaling and metabolic regulation in animals. In most animal cells, cytosolic pH is primarily regulated by Na^+^/H^+ ^exchangers in the plasma membrane, which export protons from the cytoplasm through passive exchange with extracellular Na^+ ^generated by Na^+^K^+^-ATPases [43]. Maintaining a distinct cytoplasmic pH is essential for optimal cellular metabolism, growth and proliferation [43,44]. Cytoplasmic acidification, for example, results in the blockage of secretion and endocytosis and prevents transition from G_0 _to S phase of the cell cycle [45-47]. Cytoplasmic acidification is also linked to induction of apoptotic pathways and has important ties to cancer [44,48]. Along with regulating bulk cytoplasmic pH, localized Na^+^/H^+ ^exchanger activity in regions of the plasma membrane also generates alkalinized pH microdomains that regulate the actin cytoskeleton and are necessary for cell polarization [49-51]. Increased Na^+^/H^+ ^exchanger activity in the growth cone of neurons compared with the cell body causes elevated local cytoplasmic pH, which results in increased polarization/extension of neurites [51]. Na^+^/H^+ ^exchanger activity is also upregulated in post-synaptic membranes during neuronal activity where it negatively regulates dendritic spine growth [52]. Increased Na^+^/H^+ ^exchanger localization to this membrane lowers the extracellular pH of the synaptic cleft, which inhibits pH-sensitive synaptic proteins that are responsible for spine growth, while at the same time alkalinizing cytoplasmic pH locally [52].

The intracellular pH of organelles varies widely. The endoplasmic reticulum, nucleus and peroxisomes lack an intrinsic pH regulatory system and are instead indirectly regulated by Na^+^/H^+ ^exchanger activity. Therefore, these organelles possess an intracellular pH of around 7.2, equivalent to that of the cytosol [39,43]. Mitochondria, in contrast, have an alkaline intracellular pH of around 8 that is maintained by the extrusion of protons across the inner mitochondrial membrane as a result of respiratory chain activity [43]. The intracellular pH of secretory and endocytic organelles becomes increasingly acidified along their pathways. For example, the luminal pH along the pathway from endoplasmic reticulum to secretory granule progressively decreases from around 7.2 to 5.5, while the difference in pH between early endosomes and lysosomes decreases from around 6.3 to 4.7 [43]. This gradual acidification is thought to result from a combination of two factors. First, the activity of V-ATPases found in these organelles may increase progressively through these pathways, resulting in higher proton accumulation and acidification [39,43]. Second, the degree of membrane permeability to protons and counter-ions may decrease progressively in these pathways, also contributing to lowering of luminal pH [43].

That intracellular pH is tightly regulated, and that a particular pH can exist as cytoplasmic microdomains in response to stimuli or through intracellular compartmentalization by organelles, implies that lipid pH biosensors such as PA could have important roles in this regulation. The work of Simons *et al. *[53] provides a tantalizing first glimpse into a probable role for pH-sensing by PA in the regulation of cell polarity in *Drosophila*. As part of a well-defined Wnt-signaling pathway, the protein dishevelled (Dsh) is recruited to the plasma membrane by frizzled (Fz) to facilitate the development of planar cell polarity, which is also important in the embryonic development of animals other than the fly [54]. Simons *et al. *performed a genome-wide RNA interference screen in *Drosophila *to identify genes required to maintain recruitment of a Dsh-green fluorescent protein fusion to the plasma membrane. Involvement of pH was shown as recruitment of Dsh was lost in Na^+^/H^+ ^exchanger knockdown mutants, which had altered cytoplasmic pH. A role for PA in this process is suggested by the binding of a basic domain of Dsh to PA *in vitro *through a cluster of basic amino acids that are also required for Dsh membrane localization *in vivo*. Consistent with the electrostatic/hydrogen-bond switch mechanism, this work [53] strongly implies that Dsh binds to deprotonated PA *in vivo*, binding that is dependent on cytoplasmic pH and the protonation state of the lipid. Dsh recruitment by PA *in vivo *was not demonstrated in this study, but it is exciting to speculate that PA has a role as a pH biosensor in the regulation of planar cell polarity in fly and other animal cells.

## Targeting phosphatidic acid effectors to membranes

Phosphatidic acid is present at low levels in virtually all cellular membranes [28,55]. However, PA effectors often localize exclusively to specific intracellular membranes [12,20,24,56]. An important question then is how is PA in a particular membrane recognized over PA in another? One obvious mechanism is for the cell to generate locally higher concentrations of PA, which facilitate effector recruitment. As mentioned earlier, regulated activation of enzymes such as PLD and DGK in a particular membrane is sufficient to provide such a signal for the recruitment of effectors such as the Raf and mTOR kinases [3]. And of course, regulating the localization of the lipid-modifying enzymes is a prerequisite. A clear example again comes from yeast, in which PLD changes its localization from being diffusely cytoplasmic in vegetative cells to localizing to prospore membranes during sporulation [21].

PA effectors can also be targeted to specific membranes through cooperative binding of the lipid with additional cofactors, such as membrane-anchored proteins or other lipids [1,23]. Opi1, for example, binds and senses changes in PA in the endoplasmic reticulum of yeast specifically by binding to the integral endoplasmic reticulum membrane protein Scs2 in addition to PA [24]. In fact, deletion of *SCS2 *results in release of Opi1 from the endoplasmic reticulum and its translocation to the nucleus, which indicates that binding to PA alone is not sufficient to retain it on the endoplasmic reticulum [57]. When the PA-binding domain of Opi1 is expressed on its own in yeast it localizes to the plasma membrane, the location of the predominant pool of PA in yeast [24,55]. Thus, Scs2 is a cofactor that tethers Opi1 to the endoplasmic reticulum, enabling Opi1 to sense PA there. Several PA effectors also contain additional binding sites for other lipids, such as the PIPs [23]. As we discuss later, this important class of signaling lipids comes in a variety of forms, with each form localized to a particular intracellular membrane [58]. The use of PIPs as cofactors should also enable tethering of PA effectors to particular membranes, but studies are needed to test this hypothesis.

The overall lipid composition of membranes is also likely to be important for targeting. First, the electrostatic/hydrogen-bond switch model predicts an important role for membrane-lipid composition on the localization of PA effectors. PE makes up around 15% of total phospholipids in yeast, but is distributed in widely varying ratios from membrane to membrane [28,55,59]. For example, the inner leaflet of the plasma membrane and the cytoplasmic leaflet of the Golgi have higher PE:PC ratios than most other intracellular membranes [7,55,59]. As described earlier, higher PE in these membranes would increase hydrogen bonding to the phosphomonoester of PA, thus increasing its overall negative charge. Second, more PE would allow better penetration of PA effectors into membranes, because of the increase in hydrophobic insertion sites caused by the conical shape of PE. The dual effects of PE will enhance the recognition of PA in these particular membranes.

Overall membrane surface charge is also likely to be a significant membrane-targeting factor. Using cationic fluorescent peptide probes to study membrane charge density, Yeung *et al. *[60] clearly demonstrated that intracellular membranes are negatively charged in varying degrees of strength. Of these, the cytosolic surface of the plasma membrane is the most negatively charged, due to the large amount of PS and PIPs [7,60]. Yeung *et al. *[60] found that their most cationic peptide probes (8+) bound predominantly to the plasma membrane, while probes of decreasing cationic charge (6+ to 2+) bound to intracellular membranes of correspondingly decreasing negative membrane surface charge. These fascinating findings raise the possibility that individual PA effectors may be fine-tuned to bind membranes of a particular net charge by the number and density of basic versus acidic residues they possess.

On the basis of the electrostatic/hydrogen-bond switch mechanism, changes in intracellular pH are likely to affect the membrane-targeting of effectors that employ all the mechanisms described above. Most significant will be the lowering of pH below the p*K_a _*of PA, which will result in stable protonation of PA and decreased effector binding. This should effectively override even substantial increases in local PA concentration due to enzyme action (for example, of PLD), as at low pH the effectors will have greatly reduced affinity for PA. Because lipid and protein composition varies between cellular membranes, and both are factors that fine-tune the p*K_a _*of PA, the effects of changes in pH on effector binding will depend on the p*K_a _*of PA in that particular membrane environment. For example, membranes that are rich in PE will lower the p*K_a _*of PA and thus reduce pH effects on effector binding over the neutral pH range, but may enhance pH effects in more acidic compartments.

## Can phosphoinositides act as pH sensors as well?

Along with PA, phosphomonoesters are found in other membrane lipids, including PIPs and certain sphingolipids [61,62]. PIPs are probably the best characterized class of membrane signaling lipids, and they regulate a multitude of important cellular processes [1,58,63]. Different PIPs are uniquely defined by phosphorylation of the inositol ring headgroup at different positions in varying combinations. PIPs have an important function as biomarkers for membrane recognition that helps define intracellular membrane compartments and localize organelle-specific activities. This is achieved through tight spatiotemporal regulation of their concentration by enzymes located in the particular membranes. Generally, PI(4,5)P_2 _is enriched in the plasma membrane, PI(4)P in the Golgi, and PI(3)P and PI(3,5)P_2 _in the endosomal system, thus enabling clear recognition of these membranes by the cellular machinery [58]. PI(4,5)P_2 _in the plasma membrane is a major regulator of endocytosis, cytoskeletal attachment and calcium release in the cell [64]. PI(4)P has important functions in the Golgi, where it is required for vesicle budding and secretion [63]. In endosomal membranes, generation of PI(3)P and PI(3,5)P_2 _acts as an important sorting signal for intracellular membrane trafficking [58].

But can PIPs act as pH sensors? The p*K*_*a *_values of the phosphomonoester headgroups of various PIPs have been measured using ^31^P-NMR in model membranes and range from around 6 to around 8 [62,65]. Of these, the most acidic is PI(4)P with a p*K_a _*of around 6.2, which should still titrate over physiological pH, but is possibly fine-tuned for more acidic compartments, consistent with its important function at the Golgi [65]. Of the polyphosphoinositides, Kooijman *et al. *[62] were able to measure the p*K*_*a *_values for the 3' and 5' phosphates of PI(3,5)P_2_, which are around 7.0 and 6.6, respectively. Similarly, PI(4,5)P_2 _has a p*K*_*a *_of around 6.5 in its 4' phosphate; the 5' phosphate could not be accurately measured, but is within the physiological range [65]. Thus, these PIPs are poised to titrate with changes in intracellular pH and will become protonated as intracellular pH drops, similarly to PA. The differences in ionization behavior of the different PIPs can be explained largely on the basis of the electrostatic/hydrogen-bond switch mechanism [62].

If PIPs are to act as pH biosensors, then their protein effectors must discriminate between the various charged forms and follow the rules of the electrostatic/hydrogen-bond switch mechanism. Canonical protein effectors bind to PIPs generally through structurally evolutionarily conserved binding domains, such as PH, PX, FYVE, ENTH and ANTH [1]. Non-canonical PIP-binding domains are also being found [66-69]. How these domains recognize these lipids varies greatly and includes combinations of 'specific' binding through distinct PIP-binding pockets, 'nonspecific' binding through electrostatic and hydrophobic interactions, and contributions from the physical properties of the membrane - for example, membrane curvature [1]. However, a common element shared with all binding domains is the requirement for electrostatic interaction with the phosphomonoesters of the lipids. Because PIPs also titrate within the physiological range, it is likely that at least some of these lipid-protein interactions will be pH-dependent as a result of the protonation state of the lipid.

But is there evidence for pH-dependent binding to PIPs? There are indeed examples, but in these cases the pH sensor is the protein rather than the lipid [70]. For example, ENTH, ANTH, and FYVE domains have been found to possess a critical pair of histidines in their PIP-binding sites, known as a 'histidine switch'. Like PA, histidines also possess a p*K_a _*within physiological range, which enables their protonation with decreasing intracellular pH [71,72]. Protonation increases the overall positive charge of the effector, which enables stronger electrostatic interaction with the PIP and thus higher affinity at low pH [71,72]. Although the protein is the sensor in this case, the histidine switch sets a precedent that small changes in ionization strengths can indeed influence effector binding. Optimistically, therefore, it seems possible that changes in pH that alter the charge state of PIPs may also regulate binding. In the simplest case, such interactions are likely to be with effectors that lack histidine switches, although a combination of both could provide additional regulation or fine-tuning.

To sum up, intracellular pH is precisely regulated in cells to ensure proper cellular physiology. Evolution has elegantly exploited the simple chemistry of the phosphomonoester to enable lipids to act as pH biosensors. The discovery that lipids act as pH biosensors provides a simple general mechanism for cells to monitor and rapidly respond to changes in pH. For example, pH changes may signal changes in cellular metabolism, response to nutrients or other growth signals, intracellular transport or even pathogenic states, such as infection and cancer. According to the electrostatic/hydrogen-bond switch mechanism presented by Kooijman *et al. *[29], the unique ability of the phosphomonoester to form hydrogen bonds with lipids and proteins is both the source of its physiological pH-sensing capabilities and also provides the mechanism for pH-dependent effector binding. In the case of PA, protein effectors have evolved to sense both the charged state of PA as well as its loosening effect on the hydrophobic property of the membrane surface, and it is these properties combined that allow specific recognition of PA over other anionic lipids in membranes. The presence of other membrane-targeting determinants in PA effectors provides additional specificity in their targeting to individual cellular membranes. An intriguing and important question that remains is whether other membrane lipids containing phosphomonoesters, such as PIPs, ceramide-1-phosphate, and diacylglycerol pyrophosphate, also function as pH biosensors. Given their physiological p*K_a _*values, these lipids undoubtedly can detect pH changes in cells. However, it remains an open question whether evolution once again has exploited electrostatic/hydrogen-bond switch chemistry for effector protein binding, thus making these lipids true pH biosensors.
